# DNA Hypermethylation Induced by L-Methionine Leads to Oligodendroglial and Myelin Deficits and Schizophrenia-Like Behaviors in Adolescent Mice

**DOI:** 10.3389/fnins.2021.659853

**Published:** 2021-04-20

**Authors:** Xianjun Chen, Nan-Xin Huang, Yong-Jie Cheng, Qi-Yan Cai, Yan-Ping Tian, Xing-Shu Chen, Lan Xiao

**Affiliations:** ^1^Department of Histology and Embryology, Institute of Brain and Intelligence, Army Medical University (Third Military Medical University), Chongqing, China; ^2^Department of Physiology, Research Center of Neuroscience, College of Basic Medical Science, Chongqing Medical University, Chongqing, China; ^3^Key Laboratory of Extreme Environmental Medicine, Ministry of Education, Department of Military Medical Geography, Army Medical Training Base, Army Medical University (Third Military Medical University), Chongqing, China

**Keywords:** DNA methylation, schizophrenia, oligodendroglia, myelin, epigenetic, behavior

## Abstract

Increasing evidence has demonstrated that in addition to dysfunction of neuronal circuitry, oligodendroglial dysfunction and/or disruption of white matter integrity are found in the brains of patients with schizophrenia. DNA methylation, a well-established risk factor for schizophrenia, has been demonstrated to cause neuronal dysfunction; however, whether dysregulation of DNA methylation contributes to oligodendroglial/myelin deficits in the pathogenesis of schizophrenia remains unclear. In the present study, by using L-methionine-treated mice, we confirmed that mice with DNA hypermethylation exhibited an anxious phenotype, impaired sociability, and sensorimotor gating deficits. Notably, DNA hypermethylation in oligodendroglial cells led to dysregulation of multiple oligodendroglia-specific transcription factors, which indicated disruption of the transcriptional architecture. Furthermore, DNA hypermethylation caused a reduction of oligodendroglial lineage cells and myelin integrity in the frontal white matter of mice. Taken together, these results indicate that DNA hypermethylation leads to oligodendroglial and/or myelin deficits, which may, at least in part, contribute to schizophrenia-like behaviors in mice. This study provides new insights into the possibility that precise modulation of DNA methylation status in oligodendroglia could be beneficial for the white matter pathology in schizophrenia.

## Introduction

Genetic alterations contribute to the high heritability of schizophrenia (SZ), and environmental factors that lead to aberrant epigenetic modifications may exacerbate the disease through dysregulation of risk genes (Shorter and Miller, 2015). DNA methylation, one of the most important epigenetic modifications in SZ (Montano et al., 2016; Sugawara et al., 2018), may stably integrate multiple layers of gene-environment interactions and lead to permanent alterations in brain function (Suderman et al., 2012). Theoretically, environmental insult-induced abnormalities in DNA methylation affect not only neurons but also oligodendroglial cells. In fact, the differences in DNA methylation between SZ patients and healthy controls are subtle compared to the differences in oligodendroglia-neurons (Mendizabal et al., 2019), highlighting the significance of abnormalities in cell type-specific DNA methylation in SZ. Aberrantly methylated DNA regions are clustered in multiple neuronal genes involved in dopaminergic/GABAergic signaling and synaptic transmission (Hannon et al., 2016; Rizzardi et al., 2019); however, whether and how aberrant DNA methylation can interfere with oligodendroglial development in the pathogenesis of SZ remains unclear.

Accumulating evidence from neuropathological, neuroimaging, and genetic studies has demonstrated that oligodendroglial defects and disruption of white matter integrity occur in SZ (Hof et al., 2003; Tkachev et al., 2003; Stark et al., 2004; Nave and Ehrenreich, 2014; Chen et al., 2018). Disruption of myelin integrity may occur in white matter tracts (e.g., the inferior longitudinal fasciculus) even before the onset of SZ (Friedman et al., 2008; Yao et al., 2013). In addition, rodent models with impaired oligodendroglial development and myelin deficits exhibit various phenotypes reminiscent of SZ (Poggi et al., 2016; Chen et al., 2020), indicating that oligodendroglial and myelin deficits could contribute to the pathogenesis of SZ. Moreover, dysregulation of multiple oligodendroglial-specific genes, such as Sox10, PLP1, Olig1, and Olig2, has been found in the brains of SZ patients (Nicolay et al., 2007; Liu et al., 2015; Robinson et al., 2016), indicating that epigenetic dysregulation of candidate genes in oligodendroglia could contribute to the white matter deficits observed in SZ.

The transition from oligodendroglial progenitor cells (OPCs) to mature oligodendrocytes is characterized by the dynamic expression of specific transcription factors, which, in turn, could be controlled by epigenetic regulation, such as DNA methylation (Emery and Lu, 2015; Moyon and Casaccia, 2017). A previous study found that maintaining a certain level of DNA methylation is necessary for oligodendroglial development (Moyon et al., 2016); however, substantial attention should also be paid to DNA hypermethylation. For example, hypermethylation of Sox 10, a well-established oligodendroglia-specific risk factor, was shown to be correlated with oligodendrocyte dysfunction in the brains of SZ patients (Iwamoto et al., 2005, 2006). Therefore, we hypothesized that aberrant DNA hypermethylation may interfere with oligodendroglial/myelin development and contribute to the pathogenesis of SZ.

To test our hypothesis, we employed a mouse model of DNA hypermethylation induced by L-methionine (Met) treatment. First, we examined mouse behaviors in the open field test, social interaction test and pre-pulse inhibition (PPI) test. Then, the expression of multiple transcription factors crucial for oligodendroglial development was assessed in Met-treated mice. Finally, we analyzed the development of oligodendroglial lineage cells, as well as myelin ultrastructure in the frontal white matter in mice after Met treatment.

## Materials and Methods

### Animals

C57BL/6 male mice were obtained from the Experimental Laboratory Animal Center of Third Military Medical University. Met was administered as described previously (Dong et al., 2005). Briefly, Met (Sigma-Aldrich, Cat: M9625) was dissolved in saline (0.9% NaCl). To avoid the biased influence of estrogen on DNA methylation (Dumasia et al., 2017), the adolescent male mice (6 weeks, 17–20 g) were chosen to receive twice-daily subcutaneous injections of saline vehicle (control group, Ctl) or Met (5.2 mmol/kg) for 2 weeks.

### Immunohistochemistry

Immunofluorescence staining was performed as previously described (Chen et al., 2020). The primary antibodies included mouse anti-CC1 (1:200, Millipore, Cat: OP80), rabbit anti-NG2 (1:200, Millipore, Cat: MAB5320), mouse anti-Olig2 (1:300, Millipore, Cat: MABN50), rabbit anti-Olig2 (1:300, Millipore, Cat: AB9610), rabbit anti-Sox10 (1:200, Abcam, Cat: ab180862), mouse anti-5mC (1:500, Abcam, Cat: ab10805), and rabbit anti-Ki67 (1:500, Thermo Fisher Scientific, Cat: MA514520). For 5mC staining, before being blocked in 5% BSA, the brain slices were treated with 70% ethanol (pre-cooled on ice) for 5 min and then treated with 1.5 M HCl for 30 min at room temperature.

### Fluorometric TUNEL Analyses

Brain slices were prepared as described in our previous study (Chen et al., 2020). Then, apoptosis was assessed in free-floating sections by using the DeadEnd^TM^ Fluorometric TUNEL System following the manufacturer’s protocol (Promega, Cat: G3250). After TUNEL staining, the slices were incubated with primary antibody (Olig2, 1:300, Millipore, Cat: AB9610) overnight at 4°C (five animals for each group, *n* = 5).

### Image Acquisition and Analysis

Immunofluorescence images were taken from frontal white matter (forceps minor of the corpus callosum) with a confocal microscope (FV3000, Olympus) at excitation wavelengths appropriate for Alexa Fluor 488 (488 nm), Alexa Fluor 568 (568 nm) and DAPI. For quantification of 5mC fluorescence intensity, a single focal plane was scanned under the same conditions as previously reported (Chen et al., 2019). At least three representative images (20×) were randomly acquired for each mouse. Quantification of the fluorescence intensity (*n* = 3) and cell counting (*n* = 4∼5) were performed using CellSens Dimension Desktop software (Olympus).

### Bisulfite Sequencing

Genomic DNA was extracted from control and Met treated mice brain tissues using Genomic DNA kit (TIANGEN, Cat: DP304), following by EpiTect Bisulfite kit (QIAGEN, Cat: 59104). Bisulfite-treated DNA was used for PCR amplification using the specific primers: forward, TTAGAGGTGGAGTTGAGTTTTGTG; reverse, TACCACCTATACCCACACCAT. The amplification products were cloned into T-Vector pMD^TM^19 (Takara, Cat: 3271) and then transformed in DH5α cells for clonal analyses. Each individual clone was sequenced by Sangon Company. The potential CpG island of Sox10 was predicted by MethyPrimer and the methylation status of the Sox10 CpG islands was determined (*n* = 3).

### Identification of Putative Transcription Factor-Binding Sites

JASPAR 2020^1^, a newest database of known transcription factor binding sites from the experiment-based literature (Fornes et al., 2020), was used to predict putative transcription factors binding to the human genes promoter (1000 bp upstream of the transcription start site). Relative profile score threshold was set as 85%.

### Transmission Electron Microscopy

Transmission electron microscopy was performed as previously described (Chen et al., 2020) (*n* = 3). Briefly, cubes of frontal white matter tissues were rinsed with PBS, post-fixed in 1% OsO4 in PBS for 2 h, counterstained with uranyl acetate, dehydrated in a graded ethanol series, infiltrated with propylene oxide, and embedded in Epon. Ultrathin sections were generated by an ultramicrotome (LKB-V, LKB Produkter AB, Bromma, Sweden) and were viewed with a transmission electron microscope (TECNAI10, Philips). The G-ratios of myelinated fibers were calculated as the ratio of the diameter of the axon to the diameter of the axon with the myelin sheath.

### RNA Extraction and Analysis

Total RNA was isolated using TRIzol reagent (Invitrogen). Reverse transcription and real-time PCR were performed as previously described (Chen et al., 2020). GAPDH was used as the loading control (*n* = 3). The primers are listed in Table 1.

**TABLE 1:** Primers for RT-qPCR.

Name	Forward primer	Reverse primer
OLIG1	ACCAACGTTTGAGCTTGCTT	GGTTAAGGACCAGCCTGTGA
OLIG2	AGCAATGGGAGCATTTGAAG	CAGGAAGTTCCAGGGATGAA
MBP	ATCCAAGTACCTGGCCACAG	CCTGTCACCGCTAAAGAAGC
SOX10	TGGACCACCGGCACCCAGAA	CGTGGGCAGAGCCACACCTG
ID2	ACCCGATGAGTCTGCTCTAC	CTGGTTCTGTCCAGGTCTCT
ID4	GTTCACGAGCATTCACCGTA	AAGGTTGGATTCACGATTGC
NKX2.2	GACACCAACGATGAAGACGG	TGCTGTCGTAGAAAGGGCTC
MAG	GGACCCCATCCTTACCATCT	CGGGTTGGATTTTACCACAC
PLP	CTGGCTGAGGGCTTCTACAC	GACTGACAGGTGGTCCAGGT
GAPDH	ACCCAGAAGACTGTGGATGG	CACATTGGGGGTAGGAACAC

### Behavioral Tests

The mice were housed in a controlled environment (approximately 25°C), provided free access to food and water and maintained on a 12 h/12 h day/night cycle. After each experiment, all the apparatuses were wiped clean with 70% ethanol to remove traces of the previous trial. For all behavioral experiments, the investigators were blinded to the treatment groups.

#### Open Field Test

The open-field test was performed as previously described (Chen et al., 2020) (*n* = 14). A plastic open-field chamber (50 × 50 × 50 cm; Biowill, China) was used. The mice were placed in the chamber, and their activity was recorded with a digital video camera (Digital CCD Camera, Sony). The total distance traveled, distance traveled in the center, and ratio of distance traveled in the center to total distance traveled over 10 min were recorded during the testing period.

#### Social Interaction Test

The social interaction test was conducted as previously described with slight modification (Clapcote et al., 2007) (*n* = 10). Two identical plastic square cylinders (each 8 cm in diameter and 12 cm tall) were placed in opposite corners of a box (50 cm × 50 cm × 50 cm). The subject mouse was first placed in the center of the box. After a 10 min adaptation period in which the subject was free to explore each cylinder, a stranger mouse was placed in one of the cylinders. The number of contacts with and the time spent exploring each cylinder by the subject mouse over 10 min were measured.

#### Pre-pulse Inhibition (PPI)

Pre-pulse inhibition was tested using a sound-attenuating chamber (Med Associates, Startle Reflex System ENV-022s, United States) according to our previous study (*n* = 8). The PPI test consisted of 30 trials: pulse-alone trials (120 dB, 40 ms), pre-pulse + pulse trials and no stimulus trials. The pre-pulse + pulse trials consist of a pre-pulse noise stimulus (75 dB, 20 ms) followed by pre-pulse onset (a startling pulse, 120 dB, 40 ms) 100 ms later. PPI was calculated as [1–(startle amplitude on pre-pulse trial)/(startle amplitude on pulse-alone)] × 100%.

### Statistics

Statistical analyses were performed using GraphPad Prism 5 software (GraphPad Software Inc.). For comparisons between two groups, we used paired-sample *t*-tests or independent *t*-tests when necessary. For independent *t*-tests, Welch’s correction was applied when the variance was unequal. The results were considered to be significant at *P* < 0.05.

## Results

### Mice With DNA Hypermethylation Exhibited Schizophrenia-Like Behaviors

**FIGURE 1:**
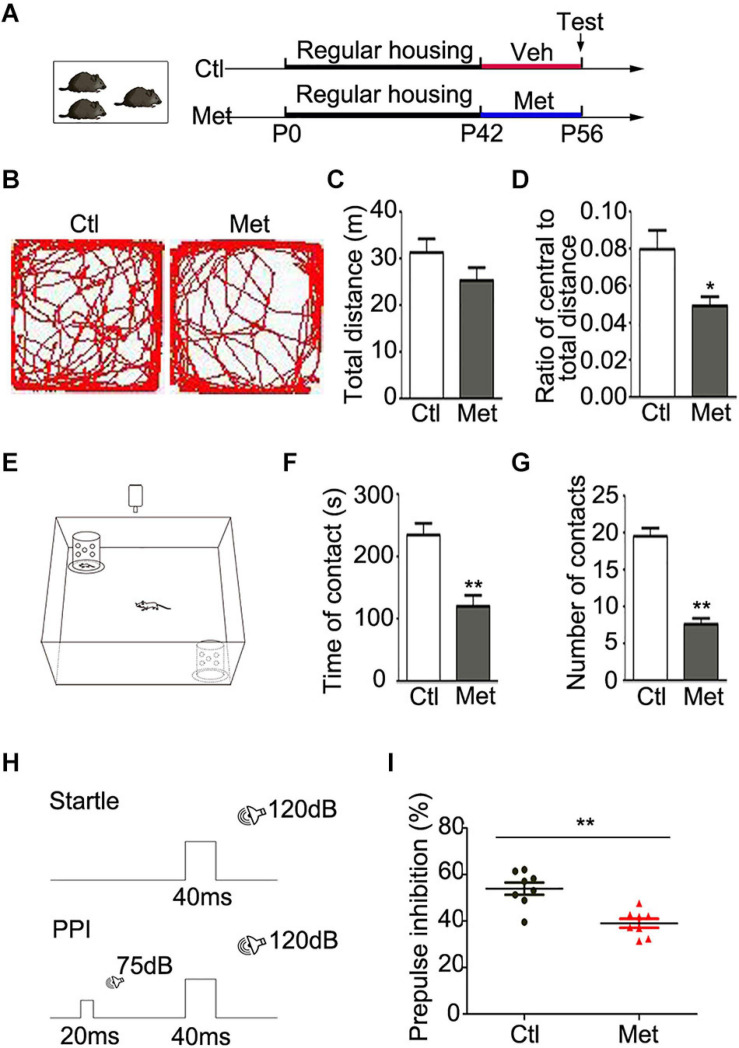
Analyses of behavioral changes in mice after Met treatment. **(A)** The experimental paradigm of drug treatment. **(B)** Representative pictures showing the path of traveling in the open field test. The total distance **(C)** and the ratio of central/total distance **(D)** in open field were detected in vehicle-treated mice (Ctl) and Met-treated mice (Met) (*n* = 14 for each group). **(E)** The experimental paradigm of social interaction test in Ctl and Met group. The total duration of social contacts **(F)** and the number of social contacts **(G)** were detected in social interaction test (*n* = 10 for each group). **(H)** The experimental paradigm for monitoring PPI in Ctl and Met group. **(I)** The pre-pulse inhibition of acoustic startle response (*n* = 8 for each group). The data are expressed as the mean ± SEM. *P < 0.05; **P < 0.01.

To determine the functional consequence of global DNA hypermethylation, we conducted a series of behavioral tests. All the experimental mice were housed in a regular environment with 3–4 per cage and then tested after 2 weeks of vehicle or Met treatment (Figure 1A). Firstly, we assessed locomotor activity with the open-field test (Figure 1B). Met-treated mice showed intact locomotion, traveling a similar total distance as vehicle-treated mice (Figure 1C, *P* = 0.150) but exhibited an anxious phenotype, traveling a shorter distance within the central area (Figure 1D, *P* = 0.012). Secondly, the social interaction test was performed to examine the sociability of the mice (Figure 1E). To our surprise, Met-treated mice showed a shorter duration of contact with stranger mice (Figure 1F, *P* < 0.001) and made fewer social contacts (Figure 1G, *P* < 0.001) than vehicle-treated mice, indicating that Met-treated mice showed impaired sociability.

Cognitive impairment is considered one of the core features of SZ in patients, which includes issues related to information processing, attention, learning and memory (Green, 2006). PPI, as illustrated in Figure 1H, is the most common method for quantifying information-processing deficits in SZ patients as well as rodents (Geyer and Ellenbroek, 2003). Strikingly, Met-treated mice showed a lower PPI value than control mice (Figure 1I, *P* < 0.001), which implied that the Met-treated mice exhibited sensorimotor gating deficits. Thus, mice with DNA hypermethylation showed several schizophrenia-like behaviors.

### DNA Hypermethylation Led to Dysregulation of Multiple Oligodendroglia-Specific Transcription Factors

**FIGURE 2:**
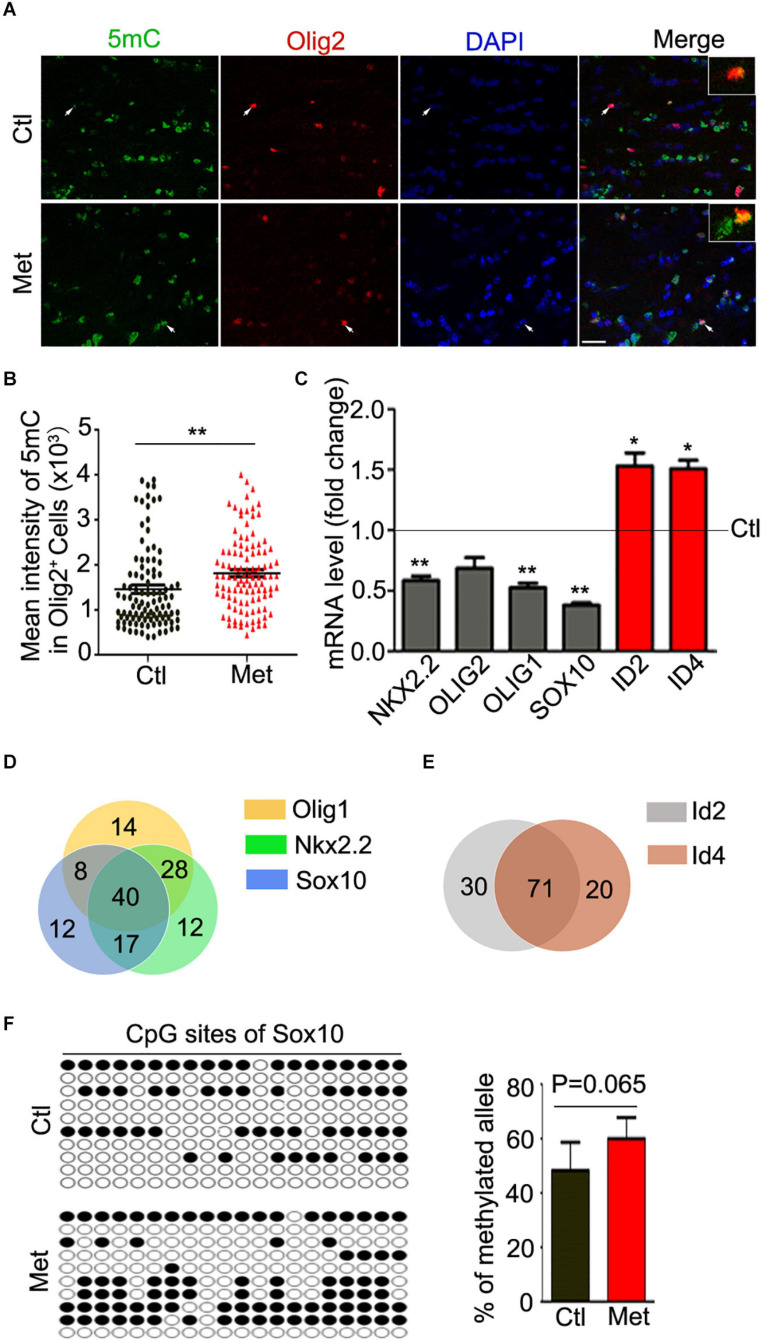
Analysis of 5mC and gene expression in oligodendroglial lineage cells after Met treatment. **(A)** Representative images showing immunofluorescence staining of 5mC and Olig2 in the frontal white matter in mice. Ctl, vehicle-treated mice. Met, Met-treated mice. White arrows indicate representative 5 mC and Olig2 duoble-labling cells, which are enlarged in white rectangle. Scale bar = 20 μm. **(B)** The bar graph shows the quantification of the mean intensity of 5mC in Olig2-staining cells (*n* = 100 cells from 5 mice in the Ctl group, *n* = 109 cells from 5 mice in the Met group). **(C)** The graph shows the quantification of NKX2.2, OLIG2, OLIG1, SOX10, ID2, and ID4 transcript levels in the frontal white matter in Met mice, which were normalized to the transcript levels in Ctl mice (dashed line) (*n* = 3 for each group). **(D)** The venn diagram shows the number of transcription factors predicted to bind to the promoters of Olig1 (yellow), Sox10 (blue), and Nkx2.2 (green). **(E)** The venn diagram shows the number of transcription factors predicted to bind to the promoters of Id2 (gray) and Id4 (red). **(F)** The dot diagram shows the methylated sites (solid points) in predicted CpG island of Sox10 promoter in Ctl and Met group. The right bar graph shows the quantification of the methylated alleles (*n* = 3 for each group). The data are expressed as the mean ± SEM. *P < 0.05; **P < 0.01.

To examine the possible contribution of oligodendroglial and myelin dysfunction to the abovementioned behavioral alterations, we first tested the effect of Met treatment on DNA methylation in oligodendroglial cells. As shown in Figure 2A, a significantly higher intensity of 5mC was detected in Olig2-positive oligodendroglial lineage cells in the frontal white matter in Met-treated mice than in vehicle-treated mice (Figure 2B, *P* = 0.003), which indicated an overall increase in DNA methylation in oligodendroglial cells. Generally, DNA hypermethylation can cause chromatin condensation and further lead to the downregulation of the expression of target genes (Baribault et al., 2018). In this study, we investigated the expression of disease-related transcriptional activators and repressors in oligodendroglia, which are highly dysregulated in schizophrenic brains. The transcripts of multiple transcriptional activators (e.g., Olig1, Olig2, Sox10, and Nkx2.2) were found to be downregulated in the Met group compared with the Ctl group. However, to our surprise, the expression of some transcriptional repressors (e.g., Id2 and Id4), which are negative regulators of oligodendroglial terminal differentiation (Emery and Lu, 2015), was significantly increased in Met-treated mice compared to vehicle-treated mice (Figure 2C).

Moreover, we searched the transcription factors binding to the promoter of above-mentioned dysregulated genes. Interestingly, only 15.6, 15.6, and 12.4% of predicted transcription factors could individually bind to the promoter of Olig1, Sox10, and Nkx2.2, respectively. The majority of transcription factors binding to the promoters of those three genes were overlayed (Figure 2D). Similarly, more than 78% of predicted transcription factors could bind to both Id2 promoter and Id4 promoter (Figure 2E). The silico analysis suggested that those dysregulated genes, especially the transcription activators, have abundant binding sites for common regulatory transcription factors, implying some similar transcription regulatory events for these genes. Moreover, a promising increase of DNA methylation at the CpG island of Sox10 promoter in oligodendroglia was confirmed (Figure 2F), which suggest that Met induced DNA hypermethylation may cause aberrations in transcriptional architecture in oligodendroglial cells.

### DNA Hypermethylation Caused Abnormalities in the Composition of Oligodendroglial Lineage Cells and Myelin Integrity

**FIGURE 3:**
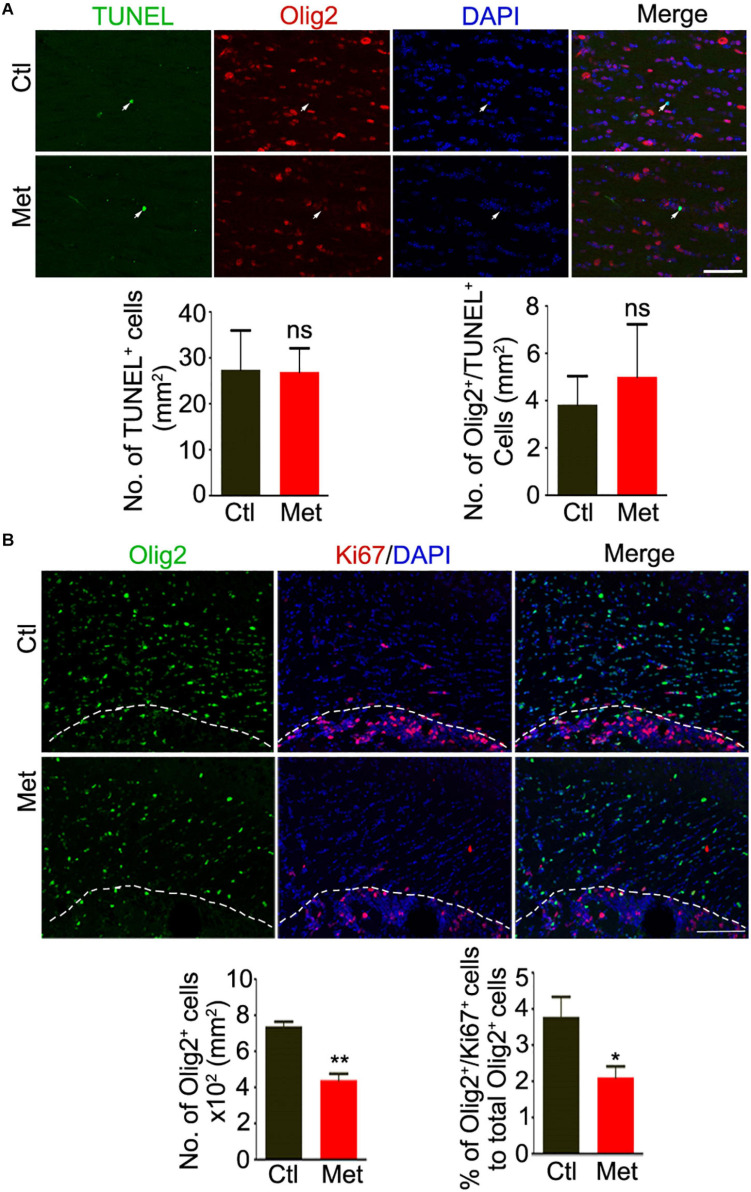
Analysis of apoptosis and proliferation of oligodendroglial lineage cells after Met treatment. **(A)** Representative images showing immunofluorescence staining of TUNEL and Olig2 in the frontal white matter of mice. White arrows indicate representative TUNEL and Olig2 duoble-labling cells. The left bar graph shows the quantification of density of TUNEL positive cells (*n* = 5 for each group). The right bar graph shows the density of Olig2/TUNEL double-labeling cells (*n* = 5 for each group). **(B)** Representative images showing immunofluorescence staining of Ki67 and Olig2 in the frontal white matter of mice. The white dashed line indicates the edge of frontal white matter. The left bar graph shows the quantification of density of Olig2 positive cells (*n* = 5 for each group). The right bar graph shows the percentage of Olig2/Ki67 double-labeling cells to Olig2 positive cells (*n* = 5 for each group). Scale bars = 50 μm. The data are expressed as the mean ± SEM. *P < 0.05; **P < 0.01; ns, no significant difference.

To directly assess how DNA hypermethylation affects oligodendroglial cells, we firstly tested whether Met treatment could lead to cell toxicity by applying TUNEL staining. As shown in Figure 3A, no significant difference was found in the density of either TUNEL-positive cells or TUNEL-Olig2 double-labeled oligodendroglial cells in the frontal white matter of brain between Ctl and Met-treated mice (Figure 3A), which excluded the toxicity of Met on oligodendroglia. However, the number of oligodendroglial lineage cells, which was indicated by Olig2 staining in the nuclei, was significantly reduced in Met-treated mice (*P* < 0.001), and the number of Ki67-Olig2 double-labeled cells was decreased either (Figure 3B, *P* = 0.029), indicating that Met-induced DNA hypermethylation may inhibit OPC proliferation.

**FIGURE 4:**
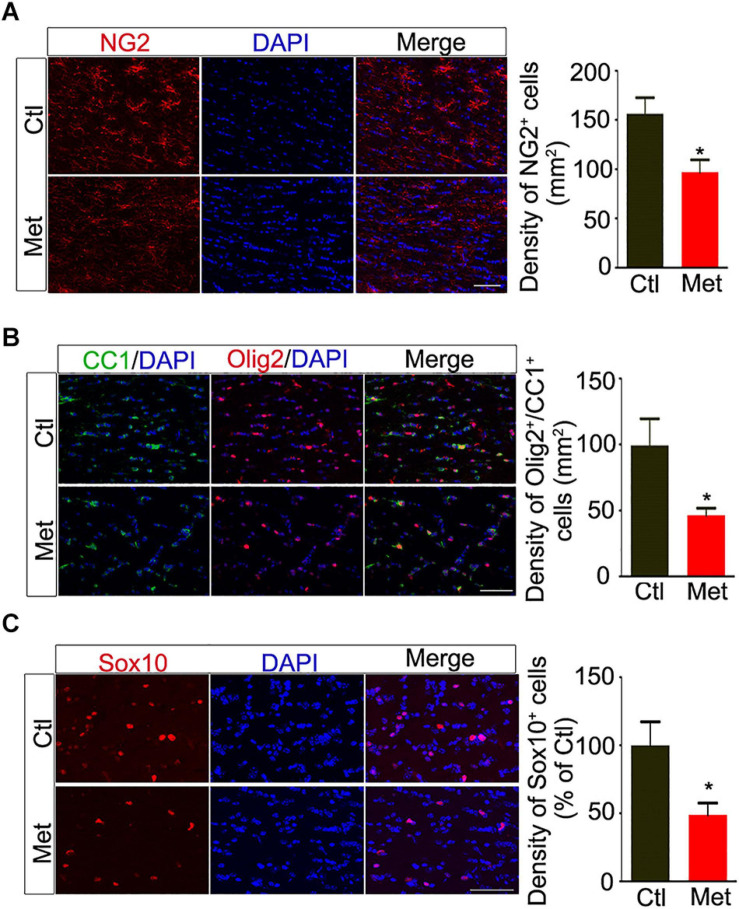
Analyses of the constitution of oligodendroglial lineage cells after Met treatment. **(A)** Representative images showing immunofluorescence staining of NG2 positive cells in the frontal white matter of Ctl and Met mice. The right bar graph shows the density of NG2 positive cells (*n* = 5 for each group). **(B)** Representative images showing immunofluorescence staining of CC1 positive cells and Olig2 positive cells in the frontal white matter of Ctl and Met mice. The right bar graph shows the density of Olig2/CC1 double-labeling cells (*n* = 5 for each group). **(C)** Representative images showing immunofluorescence staining of Sox10 positive cells in the frontal white matter of Ctl and Met mice. The right bar graph shows the density of Sox10 positive cells (*n* = 4 for each group). Scale bars = 50 μm. The data are expressed as the mean ± SEM. *P < 0.05.

**FIGURE 5:**
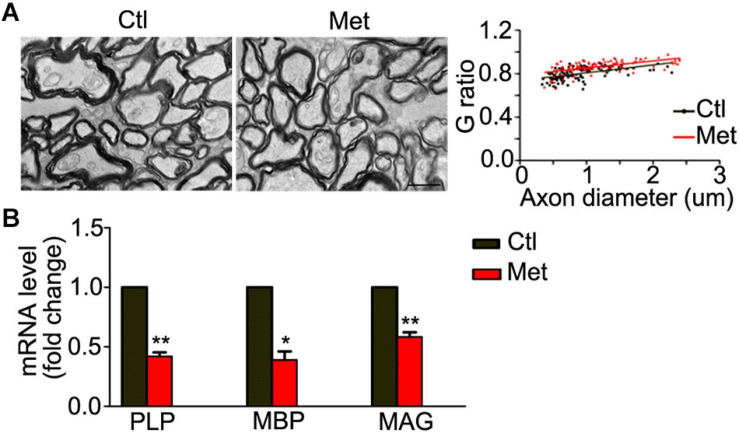
Analyses of myelin ultrastructure in the frontal white matter after Met treatment. **(A)** Representative electronic microscope pictures showing the myelin sheath in Ctl and Met group mice. The graph shows the quantification of G ratio (*n* = 3 for each group, *P* < 0.001). **(B)** The quantification of transcripts of MBP, PLP, and MAG in Met group, which is normalized to Ctl mice (*n* = 3 for each group). Scale bar = 1 μm. The data are expressed as the mean ± SEM. *P < 0.05; **P < 0.01.

Thus, we analyzed the composition of oligodendroglial cells at different stages. As shown in Figure 4A, the number of NG2 positive OPCs was significantly decreased in the Met group compared to the Ctl group (Figure 4A, *P* = 0.021). Meanwhile, the number of CC-1-positive mature oligodendrocytes, or SOX10 positive oligodendrocytes was significantly decreased in the Met group as compared to the Ctl group (Figures 4B,C). These results indicated that DNA hypermethylation may cause a reduction of oligodendroglial lineage cells. As the mature oligodendrocytes are necessary for myelination in the central nervous system, we further analyzed the organization of the myelin sheath in the frontal white matter by transmission electron microscopy. As shown in Figure 5A, the myelin sheath was significantly thinner in Met-treated mice than in Ctl mice (*P* < 0.001), while no significant difference in axonal diameter was found. Moreover, expressions of several myelin genes, such as PLP, MBP, and MAG, were robustly decreased in Met-treated mice (Figure 5B). These results indicated that DNA hypermethylation caused a decrease of oligodendroglial cells and myelin integrity.

## Discussion

In this study, we found that DNA hypermethylation in oligodendroglia caused a reduction of oligodendroglial lineage cells and myelin integrity, which may have partly contributed to SZ-like behaviors in L-methionine-treated mice. Notably, global DNA hypermethylation in oligodendroglia caused dysregulation of multiple oligodendroglia-specific transcription factors, which may have shifted the balance away from oligodendroglial myelination.

From the insight of brain development, the risk age of schizophrenia onset coincides with the key period of postnatal oligodendroglial differentiation and myelination (Miller et al., 2012; Lee et al., 2014), which ranges from juvenile to young adult in mice. Therefore, we chose to mimic DNA hypermethylation within this time window (Figure 1). To understand the functional outcomes of DNA hypermethylation, we analyzed mouse behaviors and found that Met administration induced an anxious phenotype, impaired sociability and sensorimotor gating deficits (Figure 1), which are reminiscent of some key features of SZ patients. These results were consistent with the clinical finding that the psychotic symptoms of patients with SZ are exacerbated after Met administration (Cohen et al., 1974). Similar to previous findings (Wang et al., 2015), our data confirmed that treatment of mice with Met mice could be used as an ideal model to mimic SZ.

Most studies have focused on the impact of DNA hypermethylation on neuronal functions but have ignored its effect on oligodendroglia and myelination. In this study, we found that L-methionine caused global DNA hypermethylation in oligodendroglia (Figure 2). Generally, DNA methylation at promoter regions results in transcriptional repression, either by directly preventing access of transcription factors to DNA or by recruiting cofactors that regulate chromatin remodeling (Smith and Meissner, 2013; Schubeler, 2015). During oligodendroglial differentiation, the dynamics of DNA methylation include both hypermethylation and hypomethylation of various gene promoters, implying that aberrant DNA methylation alterations could have opposite effects on various genes (Moyon et al., 2016).

In our study, Met-induced DNA hypermethylation suppressed the expression of oligodendroglia-specific transcriptional activators, such as Sox10 (Figure 2), which is crucial for oligodendroglial differentiation and myelination (Emery and Lu, 2015). However, the expression of the transcriptional repressors ID2 and ID4 (Figure 2) was significantly increased after Met treatment, which implies that some other mechanisms may be indirectly involved in governing the expression of these repressors. Notably, these repressors are crucial inhibitors of oligodendroglial differentiation (Aitkenhead et al., 2002; Marin-Husstege et al., 2006). Therefore, the opposite direction of the changes in the expression of activators and repressors implies that aberrant DNA hypermethylation in oligodendroglia may affect the balance of the transcriptional architecture, which could further impair oligodendroglial differentiation through dysregulation of downstream target genes (Sun et al., 2017). Moreover, there is quite a bit of overlap with the set of transcription factors that could regulate upregulated genes and a set that regulate the downregulated genes (Figure 2), which suggest a common regulatory motif in the promoters of dysregulated genes. We found that Met treatment could directly increase the methylation status of Sox10 (Figure 2), which may directly cause the decrease of Sox10 expression and further impair oligodendroglial differentiation.

To be noted, similar to what is observed in the brains of SZ patients (Mauney et al., 2015), we found aberrations in the composition of oligodendroglial lineage cells, with a significant reduction of both OPCs and mature oligodendrocytes (Figure 4), as well as impaired myelin integrity, after Met treatment (Figure 5). Although we did not find that Met induced an increase of apoptotic oligodendroglial cells by TUNEL staining, it is still hard to exclude the possibility that the reduction of the oligodendroglial lineage cells may be mediated via non-apoptotic death mechanisms, which urges further investigation in the future study. Our data suggests a situation that oligodendroglia may exhibit high susceptibility to aberrant DNA hypermethylation during the key period of oligodendroglial differentation and myelination in postnatal brain. Interestingly, a previous study found that knockdown of Dnmt1 suppresses OPC differentiation and causes cell damage in the later phase of differentiation, while knockdown of Dnmt3a reduces OPC proliferation, suggesting that global DNA hypomethylation can impair oligodendroglial development (Egawa et al., 2019). Therefore, we deduce that proper DNA methylation status is crucial for oligodendroglial development, while both hypermethylation and hypomethylation can impair oligodendroglial development. To be noted, except for DNA methylation, some other kinds of epigenetic regulation are also important for oligodendroglial development, such as histone modifications, chromatin remodeling, microRNAs (Emery and Lu, 2015). Therefore, whether DNA hypermethylation could affect other types of epigenetic regulators need to be addressed in the future study.

Oligodendroglial/myelin deficits have been found to affect synaptic function and contribute to anxious phenotype and cognitive dysfunction (Wang et al., 2018; Chen et al., 2020), however, we cannot exclude the effect of DNA hypermethylation on neuronal functions, especially GABAergic neuronal function (Dong et al., 2005). Therefore, in future studies, the contribution of DNA methylation in oligodendroglia to the pathogenesis of SZ should be dissected through applying conditional transgenic mice, which could manipulate DNA methylation specifically in oligodendroglial cells.

Taken together, our findings provide direct evidence that DNA hypermethylation led to a reduction of oligodendroglial lineage cells and myelin integrity in the frontal white matter, which may, at least in part, contribute to behavioral alterations reminiscent of SZ. Thus, this study suggests that restoring aberrant DNA methylation levels in oligodendroglial cells could be a novel treatment strategy for white matter pathology in SZ.

## Data Availability Statement

The original contributions presented in the study are included in the article/supplementary material, further inquiries can be directed to the corresponding author/s.

## Ethics Statement

The animal study was reviewed and approved by Third Military Medical University Institutional Animal Care and Use Committee.

## Author Contributions

XC, X-SC, and LX designed the study. XC, N-XH, and Y-JC acquired and analyzed the data. Q-YC and Y-PT also analyzed the data. XC, X-SC, and LX wrote the manuscript, which all other authors reviewed. All authors consent to participate and approved the final version for publication.

## Conflict of Interest

The authors declare that the research was conducted in the absence of any commercial or financial relationships that could be construed as a potential conflict of interest.

## Footnotes

1 http://jaspar.genereg.net/
